# Genome-wide identification, evolution, and expression analysis of *MLO* gene family in melon (*Cucumis melo* L.)

**DOI:** 10.3389/fpls.2023.1144317

**Published:** 2023-02-24

**Authors:** Taifeng Zhang, Nan Xu, Sikandar Amanullah, Peng Gao

**Affiliations:** ^1^ Key Laboratory of Biology and Genetic Improvement of Horticulture Crops (Northeast Region), Ministry of Agriculture and Rural Affairs, Harbin, China; ^2^ College of Horticulture and Landscape Architecture, Northeast Agricultural University, Harbin, China

**Keywords:** *Cucumis melo* L., genome-wide, *MLO* gene family, expression analysis, gene clone

## Abstract

Powdery mildew (PM) is one of the main fungal diseases that appear during the cultivation of the melon fruit crop. Mildew Resistance Locus “O” (MLO) is known as a gene family and has seven conserved transmembrane domains. An induced functional loss of a specific *MLO* gene could mainly confer PM resistance to melons. However, the genomic structure of *MLO* genes and its main role in PM resistance still remain unclear in melon. In this study, bioinformatic analysis identified a total of 14 *MLO* gene family members in the melon genome sequence, and these genes were distributed in an uneven manner on eight chromosomes. The phylogenetic analysis divided the *CmMLO* genes into five different clades, and gene structural analysis showed that genes in the same clade had similar intron and exon distribution patterns. In addition, by cloning the *CmMLO* gene sequence in four melon lines, analyzing the *CmMLO* gene expression pattern after infection, and making microscopic observations of the infection pattern of PM, we concluded that the *CmMLO5* (*MELO3C012438*) gene plays a negative role in regulating PM-resistance in the susceptible melon line (Topmark), and the critical time point for gene function was noticed at 24 and 72 hours after PM infection. The mutational analysis exhibited a single base mutation at 572 bp, which further results in loss of protein function, thus conferring PM resistance in melon. In summary, our research evidence provides a thorough understanding of the *CmMLO* gene family and demonstrates their potential role in disease resistance, as well as a theoretical foundation for melon disease resistance breeding.

## Introduction

Melon (*Cucumis melo* L.) is an economically important plant with excellent nutritional value (Amanullah et al., 2018; Amanullah et al., 2021). It is a widely distributed fruit crop with a long history of cultivation and has been observed to be susceptible to powdery mildew (PM), especially late in the growing season (Zhang et al., 2013). The pathogens proliferate and spread rapidly in plant parts after the PM infection (Li et al., 2017). In general, white, powdery bacteria cover the leaves, petioles, and stems of infected plants on both sides. In the later stage of infection, the leaves gradually turn yellow, die, and fall off, exposing the fruit to sunlight, which will eventually lead to the overall death of the plant late in growth, seriously affecting the quality and yield of melon (Kuzuya et al., 2003). Mainly, *Podosphaera xanthii (Px)* and *Golovinomyes cichoracearum (Gc)* are the two main fungi that are mainly responsible for the occurrence of PM disease in Cucurbitaceae crops (Křístková et al., 2009).

The Mildew Resistance Locus “O” (MLO) is a gene family unique to plants (Kim et al., 2002). It exists in many plants but is also known as an important member of the *MLO* gene, which plays a negative regulatory role in the process of plant disease resistance, which is equivalent to the “disease susceptibility” gene in plants to a certain extent. This gene was first discovered in barley, and mutants of this gene produce broad-spectrum resistance to barley PM (Schultheiss et al., 2002). Numerous studies have shown that the *MLO* gene has dual functions that are especially involved in a negative regulation of plant disease resistance and a negative regulation of plant leaf cell death (Büschges et al., 1997; Piffanelli et al., 2002). The previously published studies believed that the MLO protein has a complete set of seven transmembrane domains (Büschges et al., 1997; Devoto et al., 1999; Kim et al., 2002). Currently, it has been found that the number of MLO protein transmembrane domains varies in both lower and higher plants. In addition, MLO proteins with <5 transmembrane domains are usually found in lower plants, while MLO proteins with 6–8 transmembrane domains are usually found in higher plants (Chen et al., 2014; Zhang et al., 2018). Some studies have shown that there are 12 to 19 members of the *MLO* gene family in Arabidopsis, grape, rice, peach, woodland strawberry, and tobacco (Devoto et al., 2003; Feechan et al., 2008; Liu and Zhu, 2008; Pessina et al., 2014; Appiano et al., 2015).

In the past few decades, an extensive study of some MLO proteins related to PM resistance has been performed and successfully employed in breeding mechanism of disease resistance. Freisleben (Freisleben and Lein, 1942) treated barley (*Hordeum vulgare*) variety ‘Haisa’ by X-ray to induce mutagenesis and discovered the first mutant MLO resistant to barley PM. In 1997, further studies revealed that wheat (*Triticum aestivum*) was resistant to PM when the *MLO* gene had mutated into *mlo* (Büschges et al., 1997; Panstruga, 2005b). At present, the *MLO* gene has been implicated in PM sensitivity in *Hordeum vulgare*, *Solanum lycopersicum*, *Arabidopsis thaliana*, *Oryza sativa*, and *T. aestivum* (Elliott et al., 2002). In addition, three mutants, *Hvmlo* (Shirasu et al., 1999), *SImlo* (Bai et al., 2008), and Atmlo2/6/12 triple mutants were found to be resistant to PM (Consonni et al., 2006). In recent years, extensive studies of *MLO* genes revealed that mutations of one or more specific *MLO* genes in *Vitis vinifera*, *Petunia hybrida*, *Pisum sativum*, *Capsicum annuum*, and *T. aestivum* were found to cause broad-spectrum resistance to PM (Humphry et al., 2011; Pavan et al., 2011; Zheng et al., 2013; Wang et al., 2014; Jiang et al., 2016; Pessina et al., 2016), because there is a possibility that PM requires MLO proteins to invade host plant cells (Panstruga and Schulze-Lefert, 2003; Panstruga, 2005b). Therefore, deletion or mutation of the *MLO* gene can cause PM spores to not enter the plant cell wall (Panstruga, 2005b).

In addition, MLO protein plays a negative regulatory role in two independent osmotic PM resistance pathways (Underwood and Somerville, 2008). The synaptic fusion protein PEN1/ROR2 of the first pathway is thought to play a role in regulating vesicle transport (Collins et al., 2003); the direct interaction between MLO and ROR2 was found in yeast hybrid experiments (Panstruga, 2005a); Glucohydrolase PEN2 and ABC transporter PEN3 are involved in another pathway and produce and secrete mycotoxins (Lipka et al., 2005; Stein et al., 2006). Some studies found that the N-terminal of MLO protein was located outside of the cell; moreover, the C-terminal was located inside the cell, and there is a calmodulin-binding domain (CaMBD) of 10–15 amino acids away from the 7^th^ transmembrane domain (Xu and Heath, 1998; Kim et al., 2002; Bhat et al., 2005). It was also proposed that two other conserved domains at the C-terminal of an MLO protein might be related to the sensitivity of a plant to PM (Panstruga and Schulze-Lefert, 2003; Feechan et al., 2008). However, Ca^2+^ also plays an important role in regulation of the plant defense response, and a large number of studies have found that the intracellular Ca^2+^ level increases rapidly when plants are infected by pathogens (Xu and Heath, 1998; Blume et al., 2000). Calmodulin is an important Ca^2+^-binding protein that plays an important role in the calcium signaling pathway (Yang and Poovaiah, 2003). At present, MLO proteins have been identified in numerous crops (*Hordeum vulgare*, *Oryza sativa*, *Brachypodium distachyon*, *Medicago truncatula*, *Cajanus cajan*, and *Phaseolus vulgaris*), and have been found to contain CaMBD (Liu and Zhu, 2008; Ablazov and Tombuloglu, 2016).

In this study, we carried out a systematic and comprehensive bioinformatics analysis of *CmMLO* gene family members in the melon genome database. Nucleotide sequence information, physicochemical properties, phylogenetic relationships, and expression characteristics of the *CmMLO* gene family were analyzed, which provided the theoretical basis for a further methodical study of the function of each member of the *CmMLO* gene family.

## Materials and methods

### Plant materials and inoculation treatment

**Figure 1:**
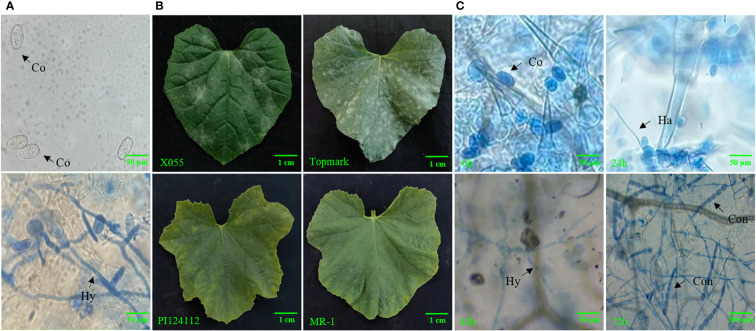
**(A)** Microscopic observation of morphology of PM-susceptible line (Topmark) infected with *Podosphaera xanthii*, **(B)** Phenotypic characteristics of PM-susceptible lines (X055 and Topmark) and PM-resistance lines (PI124112 and MR-1), **(C)** Fungal morphology of PM-susceptible line (Topmark) infected with by *P. xanthii* race 1 at different time interval. Co, conidium; Ha, haustorium; Hy, hyphae; Con, conidiophore.

A total of four types of melon lines “PM-resistant lines (MR-1 and PI124112) and PM-susceptible lines (X055 and Topmark)” were selected for experiment materials (**Figure 1**), and identification of physiological races of PM was done using thirteen general international host identification Cucurbitaceae (Iran H, Topmark, Védrantais, PMR 45, PMR 5, WMR 29, Edisto 47, PI 414723, MR-1, PI 124111, PI124112, PMR 6, and Nantais Oblong). The above materials were provided by the Laboratory of Molecular Genetics and Breeding in Melon, Northeast Agricultural University, Harbin, Heilongjiang, China. All the test materials were grown in nutrient bowls (9.3 cm in diameter, 9 cm deep) in a controlled environment of greenhouse and kept sterile for several weeks before infection treatment. When the plants reached the three-leaf stage, PM fungal spore suspension spray was used for artificial inoculation, and the concentration of inoculated spores was 1×10^6^/mL. Three separate biological replicates were used at each time interval throughout the experiment. Plant leaf tissues were collected at 0, 24, 48, and 72 h after inoculation, snap-frozen in liquid nitrogen, and stored at −80°C prior to the further experiments.

### Microscopic observation of PM on melon leaves

The hyphal development was visualized using the Trypan Blue staining technique (Koch and Slusarenko, 1990). In brief, the PM-infected melon leaves were soaked in Trypan Blue staining solution and immediately heated in 100 °C water for 2–5 min. After allowing the solution to cool down to room temperature, the staining solution was discarded and the leaves were decolored using a 2.5 mg/ml chloral hydrate solution, which was replaced every 24 hours until the leaves were completely decolored.

### Genome-wide identification of the *MLO* gene family

Firstly, the publicly available genomic data of melon (v3.5.1), watermelon, and cucumber were obtained from the Cucurbitaceae genomic database (http://cucurbitgenomics.org/), the genomic dataset of Arabidopsis was obtained from the The Arabidopsis Information Resource (TAIR) website (https://www.arabidopsis.org/), and bio-informatics analysis were performed as reported earlier (Yang et al., 2022). A total of 15 MLO protein sequences in *A. thaliana* were obtained from the TAIR database, and all the melon proteins were compared with the 15 MLO proteins in *A. thaliana* to obtain the possible gene family members and a bi-directional comparison was performed by using the TBtools software (Chen et al., 2020). Then, all non-redundant protein sequences were analyzed based on further screening, and conservative domains were analyzed to identify the MLO domain using the online website of National Center for Biotechnology Information CD Search (NCBI) (https://www.ncbi.nlm.nih.gov/Structure/bwrpsb/bwrpsb.cgi) and TBtools software. Meanwhile, a total of 14 *MLO* genes obtained from melon were respectively named (*CmMLO1*–*CmMLO14*), according to the earlier reported study (Xu et al., 2014).

### Gene structural and conserved motif analysis of the *MLO* genes

The amino acid sequences of the *MLO* gene family in melon were also analyzed by using the online analysis software Multiple Expectation Maximizations for Motif Elicitation (MEME) (http://meme-suite.org/), and ten motifs were identified for subsequent analysis. Meanwhile, a gene structure map was obtained by using the online analysis software of an upgraded gene feature visualization server, “Gene Structure Display Server (GSDS), v2.0” (http://gsds.gao-lab.org/) (Hu et al., 2015).

### Phylogenetic tree construction, chromosomal location, and collinearity analysis of the *MLO* genes

The multiple alignments of the amino acid sequences of the *MLO* gene family were analyzed using the ClustalW software (https://www.genome.jp/tools-bin/ClustalW), and defective reading-box sequences and redundant sequences were eliminated manually. The phylogenetic tree of the *MLO* gene family was constructed using the Neighbor Joining (NJ) method by using the Mega (v7.0) software (http://megasoftware.net) (Chung et al., 2015; Kumar et al., 2016); the bootstrap method with an adjusted value of 1000 was used to evaluate the phylogenetic tree (Kumar et al., 2016), and the constructed tree was enhanced graphically using the itol website (https://itol.embl.de/). The chromosomal location of the *MLO* gene family was extracted from the GFF file of melon data, and a chromosome linkage map was constructed by the TBtools software. Then, the collinearity analysis of *MLO* genes in melon, cucumber, and *A. thaliana* was performed, and Circos maps were generated.

### *MLO* genes cloning in melon

The cloning of the *MLO* genes sequences was performed for PM-resistant lines (MR-1 and PI124112) and PM-susceptible lines (X055 and Topmark), as earlier reported method (Zhang et al., 2023). The information of primer sequences for gene clone can be seen in **Supplementary Table S1**. The amplification of Polymerase Chain Reaction (PCR) was performed as follows: 1 cycle at 95°C for 5 min, followed by 30 amplification cycles at 95°C for 30 s, 50°C for 30 s, and 72°C for 90 s. Then, the amplified targeted fragments were aligned to the vector pMD18-T, and sequencing was performed at Sangon Biotech Co., Ltd.

### Expression analysis of *MLO* genes in melon in response to *P. xanthii race*

The infected leaf samples were collected from the four different time intervals of post-inoculation (0 h, 24 h, 48 h, and 72 h). Total RNA was extracted using the Trizol method, and complementary DNA (cDNA) was synthesized through reverse transcription using a ReverTra Ace qPCR RT Kit (Toyobo, Osaka, Japan). The SYBR Green I fluorescence method was used for performing the quantitative real-time polymerase chain reaction (qRT-PCR). The reaction conditions were as follows: pre-denaturation at 94°C for 1 min, denaturation at 94°C for 10 s, annealing at 55°C for 10 s, and extension at 72°C for 20 s. Finally, the obtained qRT-PCR data was processed and analyzed by the 2^−ΔΔCt^ method (Zhang et al., 2021). The information of primer sequences used for qRT-PCR can be seen in **Supplementary Table S2**.

### Statistical data analysis

A total of three biological replicates were performed for each experiment, the numerical datasets were recorded on Microsoft Excel (2016 version), and statistical analysis of descriptive (mean ± standard deviation (x ± s)) statistics was performed using the GraphPad Prism 8.0 software, respectively.

## Results

### Identification of physiological races of PM

The leaves of PM-infested melons were stained with Trypan blue, and the morphology of the pathogen was observed by light microscopy (**Figure 1**). It was found that the conidia were around 56–75 μm long and 35–43 μm wide; their shape showed an ovoid shape with a fibrous structure. It was also observed that the mycelium of PM showed linear, curved, and branched types, and that the conidiophores had a long chain structure. By observing the state, length, and width of the conidiophores presented and the presence of fibrils, it was possible to determine that the PM used in this test had the characteristics of *P. xanthii*. Using thirteen international general hosts of Cucurbitaceae as test material and infected with PM, the PM fungus studied in this trial was further identified as *P. xanthii* race 1 based on the resistance (R) and susceptibility (S) of the thirteen internationally identified hosts (**Table 1**).

**Table 1:** Powdery mildew resistance responses of hosts identification.

Host	Characterization
PMR 45	R
TopMark	S
Vedrantais	S
IranH	S
Nantais Oblong	S
Edisto 47	R
WMR 29	R
MR-1	R
PI 414723	R
PI 124112	R
PI 124111	R
PMR 5	R
PMR 6	R

S is denoting the susceptible and R is representing the resistance characterization, respectively.

### Genome-wide identification of *MLO* gene family

In total, 14 candidate genes of the *MLO* family were retrieved from the Cucurbitaceae genome database at different chromosomal locations of whole genome (**Table 2**). The amino acid (aa) sequences of *MLO* genes were analyzed by Expasy-Protparam, the longest and shortest protein sequences of the *MLO* gene family contained a maximum of 582 and a minimum of 150 amino acids, the molecular weight of *MLO* gene family members in melon ranged from 16.11–66.66 kiloDaltons (kD), the size of the isoelectric point (IP) ranged from 7.25–9.48, and the Introns were ranged from 1–16, respectively. At the same time, TMHMM online software was used to predict the transmembrane domain of the *MLO* gene family, and it was also found that the transmembrane domain of 14 *MLO* genes ranged from 2–7, respectively.

**Table 2:** Identification of fourteen *MLO* genes in melon genome.

Number	Gene ID	Chromosomal location	CDS	Protein length	Introns	TM	PI	Molecular weight/kD
*CmMLO1*	*MELO3C000169*	Chr0	450	150	1	2	8.71	16.11
*CmMLO2*	*MELO3C005038*	Chr12	588	195	4	3	9.08	21.51
*CmMLO3*	*MELO3C005044*	Chr12	1743	580	13	7	9.38	66.66
*CmMLO4*	*MELO3C007979*	Chr08	1749	582	14	7	9.34	66.37
*CmMLO5*	*MELO3C012438*	Chr10	1713	570	14	7	9.25	65.35
*CmMLO6*	*MELO3C013709*	Chr06	1719	572	14	7	8.84	64.58
*CmMLO7*	*MELO3C013761*	Chr06	1443	480	14	6	7.25	54.85
*CmMLO8*	*MELO3C016709*	Chr07	1683	560	13	7	9.1	57.23
*CmMLO9*	*MELO3C019435*	Chr06	1671	556	14	7	9.02	63.73
*CmMLO10*	*MELO3C021515*	Chr09	1662	553	16	7	8.8	64.01
*CmMLO11*	*MELO3C022486*	Chr11	1107	368	11	5	9.18	42.22
*CmMLO12*	*MELO3C023219*	Chr11	1398	465	11	7	9.48	54.87
*CmMLO13*	*MELO3C025761*	Chr11	1314	437	12	7	9.35	50.25
*CmMLO14*	*MELO3C026525*	Chr03	1629	542	14	7	8.69	61.59

### Structural analysis of the *CmMLO* gene family and prediction of conserved motif

**Figure 2:**
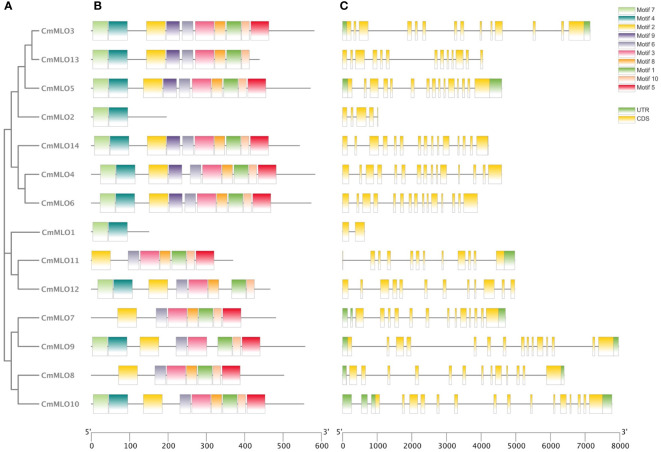
Analysis of conserved motif and gene structure of the *CmMLO* gene family. **(A)** phylogenetic tree of the *CmMLO* gene family, **(B)** *CmMLO* gene motif, **(C)** *CmMLO* gene structure.

The intron distribution provides important evidence related to the phylogenetic relationships among gene family members in plant evolution studies using the online tool “GSDS”. The analysis of gene structure showed that the structure of the *MLO* gene family was complex (**Figure 2**), and introns existed in each gene. The *CmMLO10* gene contained most of the introns, along with 16 introns, while the *CmMLO1* gene had only 1 intron. In terms of gene length, the length of the *CmMLO4* gene was significantly longer when compared to other genes. Seven gene (*CmMLO13, CmMLO2, CmMLO14, CmMLO4, CmMLO6, CmMLO1, CmMLO12*) was found without the UTR region at both ends, and one gene (*CmMLO11*) similarly lacked the UTR region at one end. Gene structural analysis showed that genes in the same clade had similar intron and exon distribution patterns. For example, four genes in the Clade VI group had the same distribution characteristics of the genetic structure.

**Figure 3:**
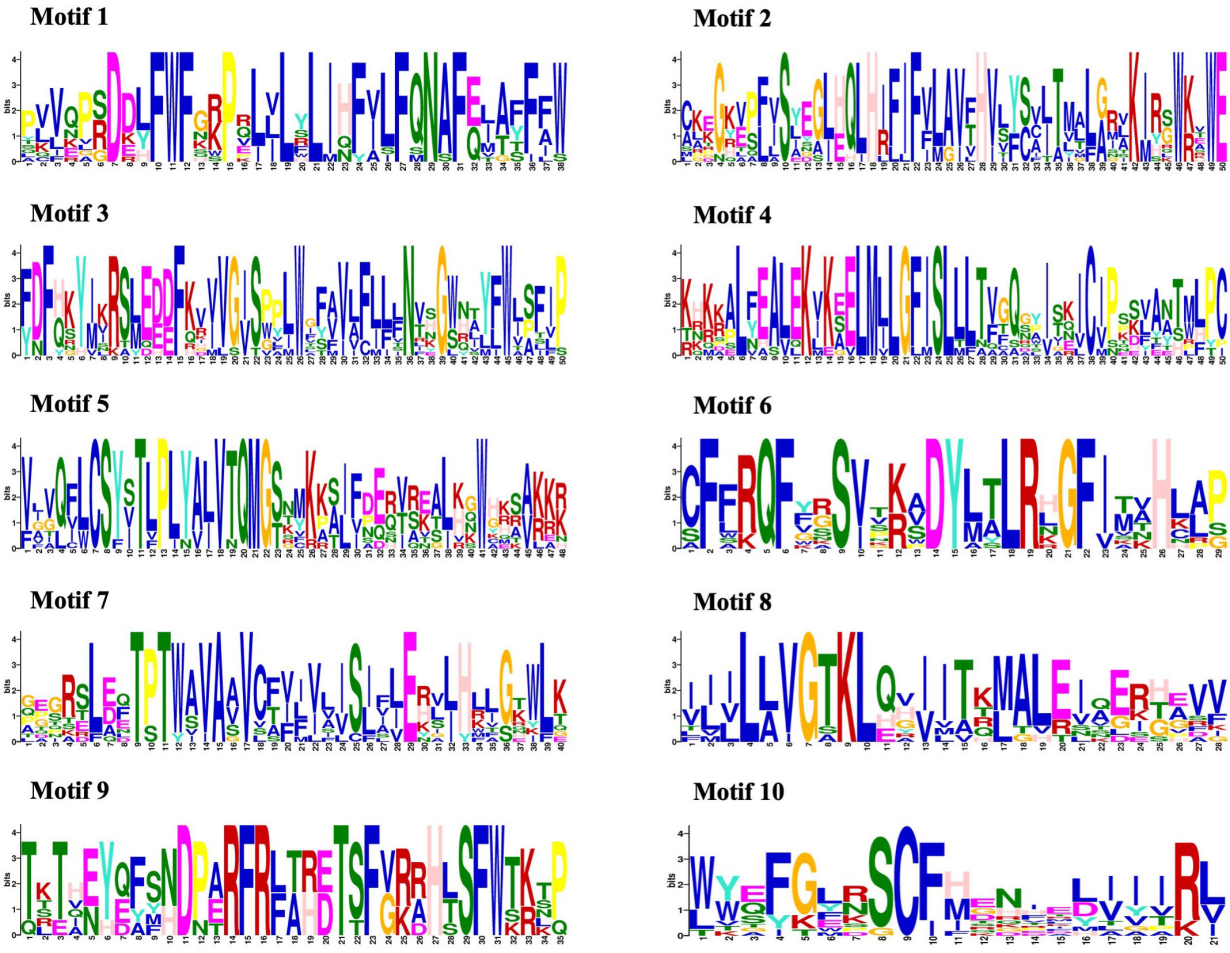
Conserved motif analysis of the CmMLO proteins.

The amino acid sequences of 14 *CmMLO* genes were analyzed by online MEME software, and ten conserved motifs were obtained. Among them, five genes had ten conserved motifs; *CmMLO11, CmMLO7, CmMLO8* lacked motif 7, and the *CmMLO1* gene and *CmMLO2* had two conserved motifs because the protein sequence of *CmMLO1* and *CmMLO2* was the shortest. The results showed that all of the genes in Clades I did not lack conserved motifs; among them, all four genes in Clade VI had lost some conserved motifs. Meanwhile, we also found that motif 5 was missing in four genes (*CmMLO2, CmMLO1, CmMLO13* and *CmMLO12*) and motif 8 was missing in three genes (*CmMLO2, CmMLO9*, and *CmMLO1*). The results suggested that these conserved motifs are relatively conserved and may play a very important role in the function of the *MLO* gene family. Finally, these findings demonstrated that 14 MLO genes in melon are relatively consistent in conserved motif conservation and have consistent location distribution among some genes. To further understand each conserved motif, we used the MEME online tool to draw a map of motifs 1–10 (**Figure 3**). The above analysis of the conserved motifs shows that motifs 1 and 4 are highly conserved and these two conserved motifs consist of 38 and 50 amino acid sites, respectively, of which motif 1 has ten completely conserved sites and motif 4 has nine completely conserved sites. The common amino acid conserved site of these two conserved motifs was leucine.

### Chromosomal location and collinearity analysis of the *MLO* genes

**Figure 4:**
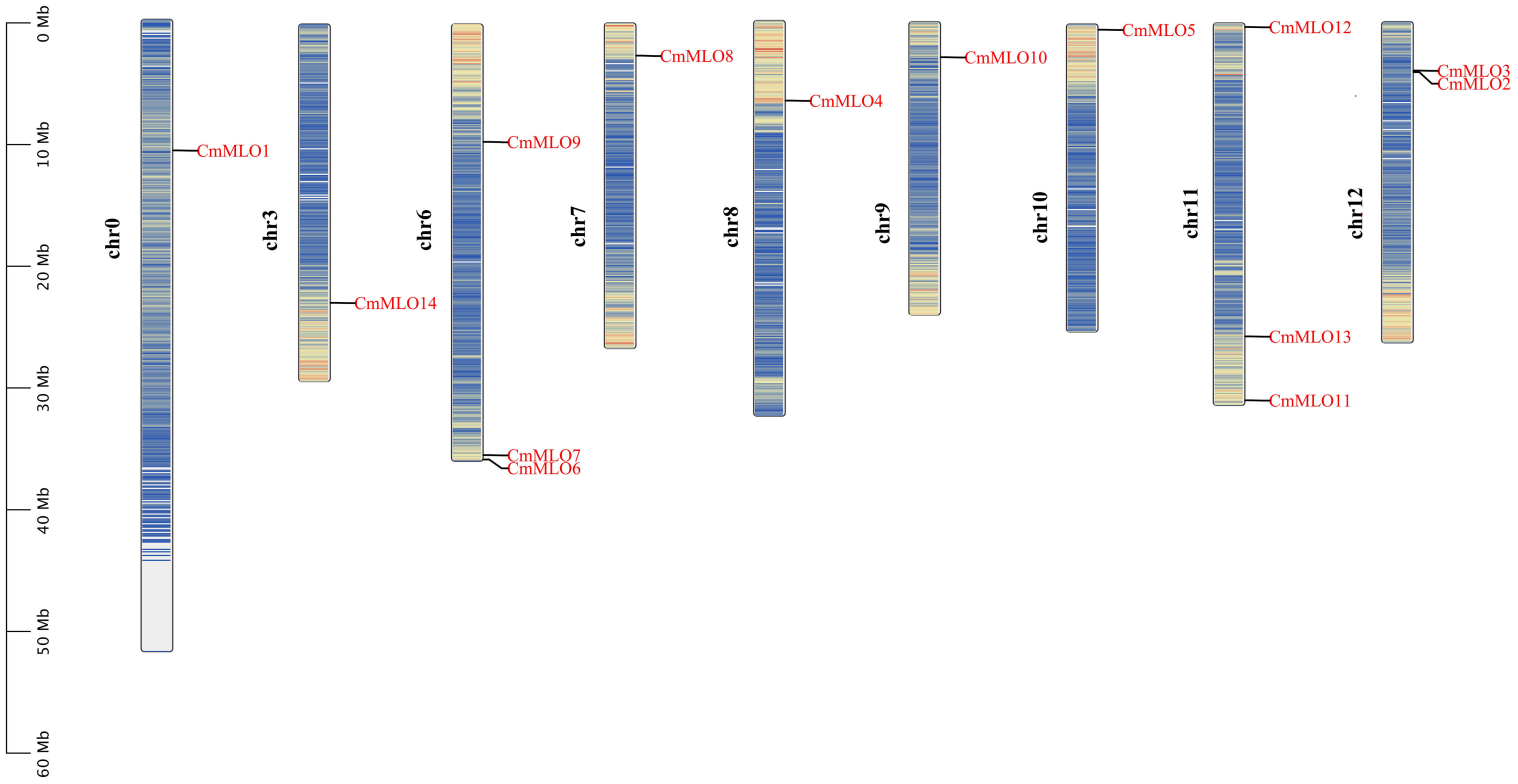
Chromosomal distribution of the *CmMLO* gene family members.

**Figure 5:**
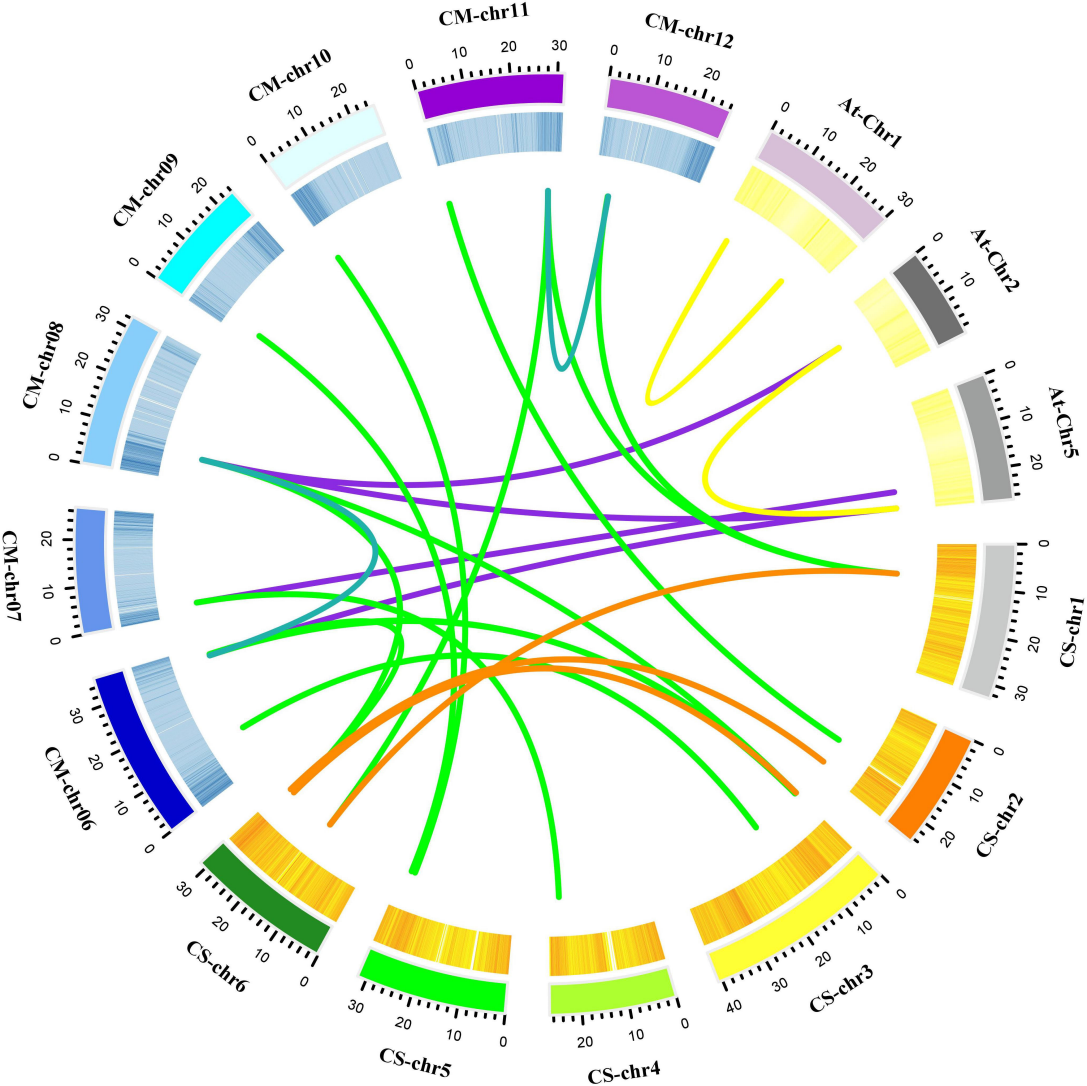
Collinearity analysis of the *MLO* gene family in melon. Different line colors indicate the following; green is showing homology between the melon and cucumber genomes; purple is showing homology between the melon and Arabidopsis genomes; blue is showing homology within the melon genome; yellow is showing homology within the Arabidopsis genome; and orange is showing homology within the cucumber genome. These gene pairs are connected by links between chromosomes.

The positions of these 14 *MLO* genes were identified on their respective chromosomes (**Figure 4**), among which the *CmMLO1* gene was located on pseudo-chromosome (Chr0) and the other genes were distributed on genetic positions of eight different chromosomes of melon genome; however, three *MLO* genes were found on two chromosomes (Chr6 and Chr11), respectively. A total of four *CmMLO* genes (*CmMLO6*, *CmMLO4*, *CmMLO13*, and *CmMLO2*) were involved in two fragment repeats; among them, *CmMLO6* and *CmMLO4* are fragments of repeating genes from the same clade. To explore the evolutionary relationship of *MLO* genes in different species (*A. thaliana*, *Cucumis melo*, and *C. sativus*), we performed collinearity analyses of genes in three plants (**Figure 5**). We found 26 pairs of duplicated fragments involving 31 genes in three species. The *CmMLO* and *CsMLO* genes were the most collinear, with 15 pairs in total. This means that these genes have changed little over time and play an important role in PM-resistance in melon and cucumber.

### Cloning of the *CmMLO* genes and bioinformatics analysis

To investigate the differences in *MLO* genes in melon lines with different PM resistance, two PM-resistant lines (MR-1 and PI124112) and two PM-susceptible lines (Topmark and X055) were selected as templates for the cloning of the *CmMLO* genes, and a total of thirteen genes were cloned (**Supplementary Figure S1**). The CDS lengths and amino acid (aa) numbers of the 13 *CmMLO* genes in the four melon lines are shown in **Table 3**. The transmembrane domain of CmMLO proteins was analyzed by using TMHMM (v2.0), and the results showed that 13 CmMLO proteins have 2–7 TM structures. However, different CmMLO proteins possess different numbers of TM structures, and the transmembrane domains of the same CmMLO protein differ significantly in different melon lines. The CmMLO1, CmMLO2, CmMLO4, CmMLO7, CmMLO10 and CmMLO11 proteins had the same number and position of TM structures in all four melon lines; there were 2, 3, 7, 6, 7, and 5 TM structures, respectively. However, CmMLO3 protein has 3 TM structures in the PM-resistant line MR-1, which is significantly different from the other three melon lines. The TM structures of the CmMLO5 protein in the PM-resistant line (MR-1) significantly differed as compared to the two PM-susceptible lines (X055 and Tompark), with only 5 TM structures in MR-1. However, the position and amount of CmMLO6 protein differed significantly between four melon lines: CmMLO12 protein has 5 TM structures in the PM-resistant line (MR-1) and 7 TM structures in all other melon lines; CmMLO13 protein has 3 TM structures in the PM-resistant line (PI124112), which was significantly different from the other three melon lines.

**Table 3:** The detailed information of CDS and protein length of the *CmMLO* genes.

Melon lines - genes	CDS	Protein	TM	Melon lines - genes	CDS	Protein	TM
MR-1 - *CmMLO1*	450	150	2	MR-1 - *CmMLO8*	1707	565	6
PI124112 - *CmMLO1*	450	150	2	PI124112 - *CmMLO8*	1707	565	6
X055 - *CmMLO1*	450	150	2	X055 - *CmMLO8*	1707	568	7
Topmark - *CmMLO1*	450	150	2	Topmark - *CmMLO8*	1707	568	7
MR-1 - *CmMLO2*	588	196	3	MR-1 - *CmMLO9*	1610	518	3
PI 124112 - *CmMLO2*	588	196	3	PI 124112 - *CmMLO9*	1610	518	3
X055 - *CmMLO2*	588	196	3	X055 - *CmMLO9*	1671	556	7
Topmark - *CmMLO2*	588	196	3	Topmark - *CmMLO9*	1671	556	7
MR-1 *- CmMLO3*	1741	565	3	MR-1 *- CmMLO10*	1662	552	7
PI 124112 *- CmMLO3*	1743	580	7	PI 124112 *- CmMLO10*	1662	552	7
X055 *- CmMLO3*	1743	580	7	X055 *- CmMLO10*	1662	552	7
Topmark *- CmMLO3*	1743	580	7	Topmark *- CmMLO10*	1662	552	7
MR-1 - *CmMLO4*	1704	567	7	MR-1 - *CmMLO11*	1107	368	5
PI 124112 - *CmMLO4*	1704	567	7	PI 124112 - *CmMLO11*	1107	368	5
X055 - *CmMLO4*	1704	567	7	X055 - *CmMLO11*	1107	368	5
Topmark - *CmMLO4*	1704	567	7	Topmark - *CmMLO11*	1107	368	5
MR-1 - *CmMLO5*	1714	559	5	MR-1 - *CmMLO12*	1397	458	5
PI 124112 - *CmMLO5*	1713	570	7	PI 124112 - *CmMLO12*	1395	464	7
X055 - *CmMLO5*	1713	570	7	X055 - *CmMLO12*	1398	465	7
Topmark - *CmMLO5*	1713	570	7	Topmark - *CmMLO12*	1398	465	7
MR-1 - *CmMLO6*	1718	550	5	MR-1 - *CmMLO13*	1314	437	7
PI124112 - *CmMLO6*	1617	530	7	PI124112 - *CmMLO13*	1313	425	3
X055 - *CmMLO6*	1635	530	5	X055 - *CmMLO13*	1314	437	7
Topmark - *CmMLO6*	1718	557	5	Topmark - *CmMLO13*	1314	437	7
MR-1 - *CmMLO7*	1443	480	6				
PI 124112 - *CmMLO7*	1443	480	6				
X055 - *CmMLO7*	1443	480	6				
Topmark - *CmMLO7*	1443	480	6				

**Figure 6:**
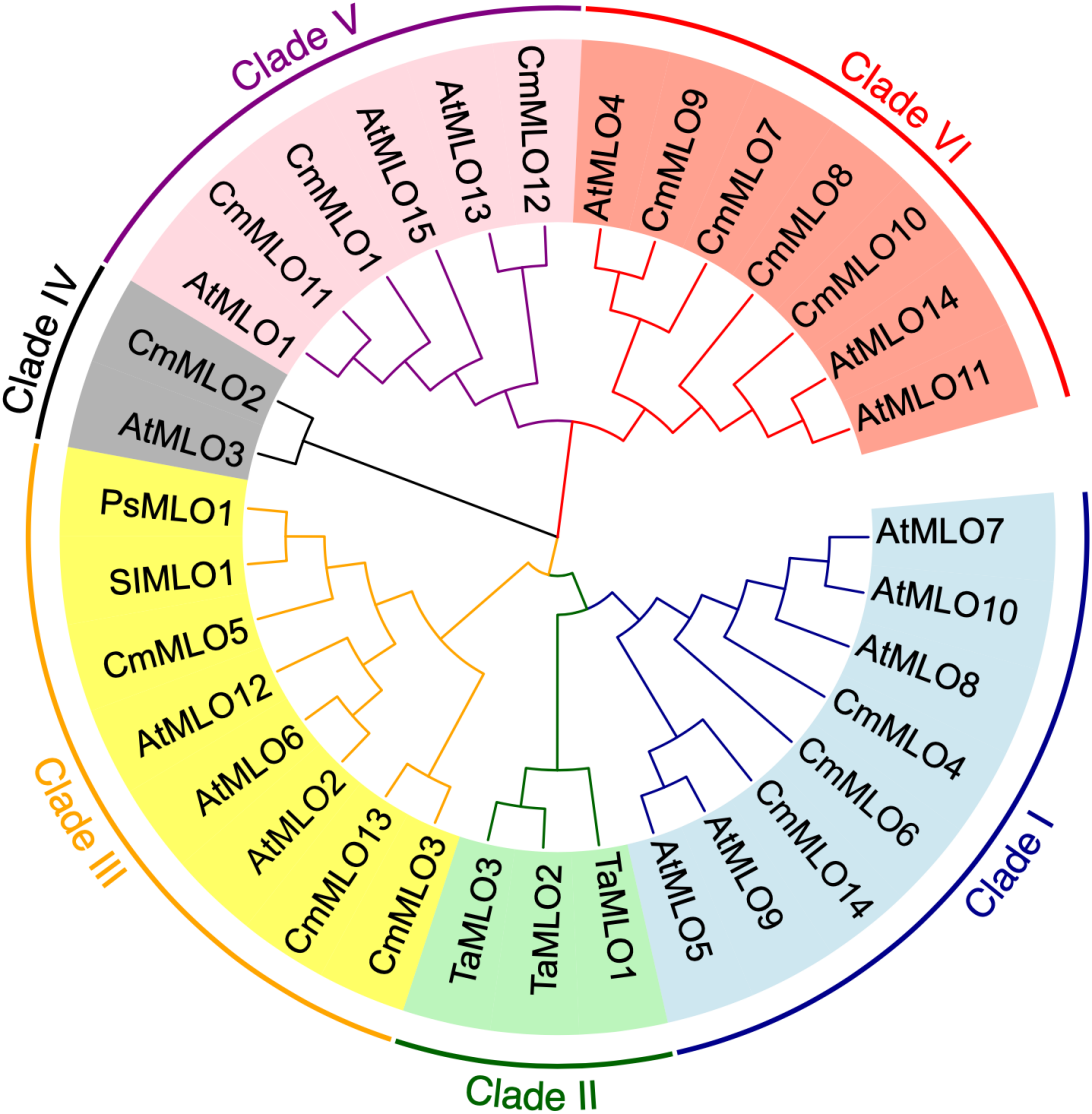
Schematic diagram of the *CmMLO* gene family in constructed phylogenetic tree.

In order to better study the *CmMLO* gene family, a phylogenetic tree was constructed by combining the CmMLO and MLO proteins of different species (Arabidopsis, wheat, tomato, and pea) (**Figure 6**). The results showed that the 14 CmMLO proteins obtained from melon genome were divided into different branches, in which CmMLO7, CmMLO9, CmMLO8, and CmMLO10 proteins were distributed with *AtMLO4*, 11 and 14 proteins in Clade VI; CmMLO1, CmMLO12, and CmMLO11 proteins are distributed with AtMLO1, 13, 15 proteins in Clade V; CmMLO6, CmMLO4 and CmMLO14 proteins are distributed with *AtMLO5*, 7, 8, 9, 10 proteins in Clade I; CmMLO13, CmMLO3 and CmMLO5 proteins were distributed with AtMLO2, 6 and 12 proteins, SIMLO1 protein, PsMLO1 protein in Clade III; CmMLO2 protein is distributed with AtMLO3 protein in Clade IV. Although the genes from the same branch are presumed to have similar roles in plant growth and development because of their high similarity of genes from the same branch.

**Figure 7:**
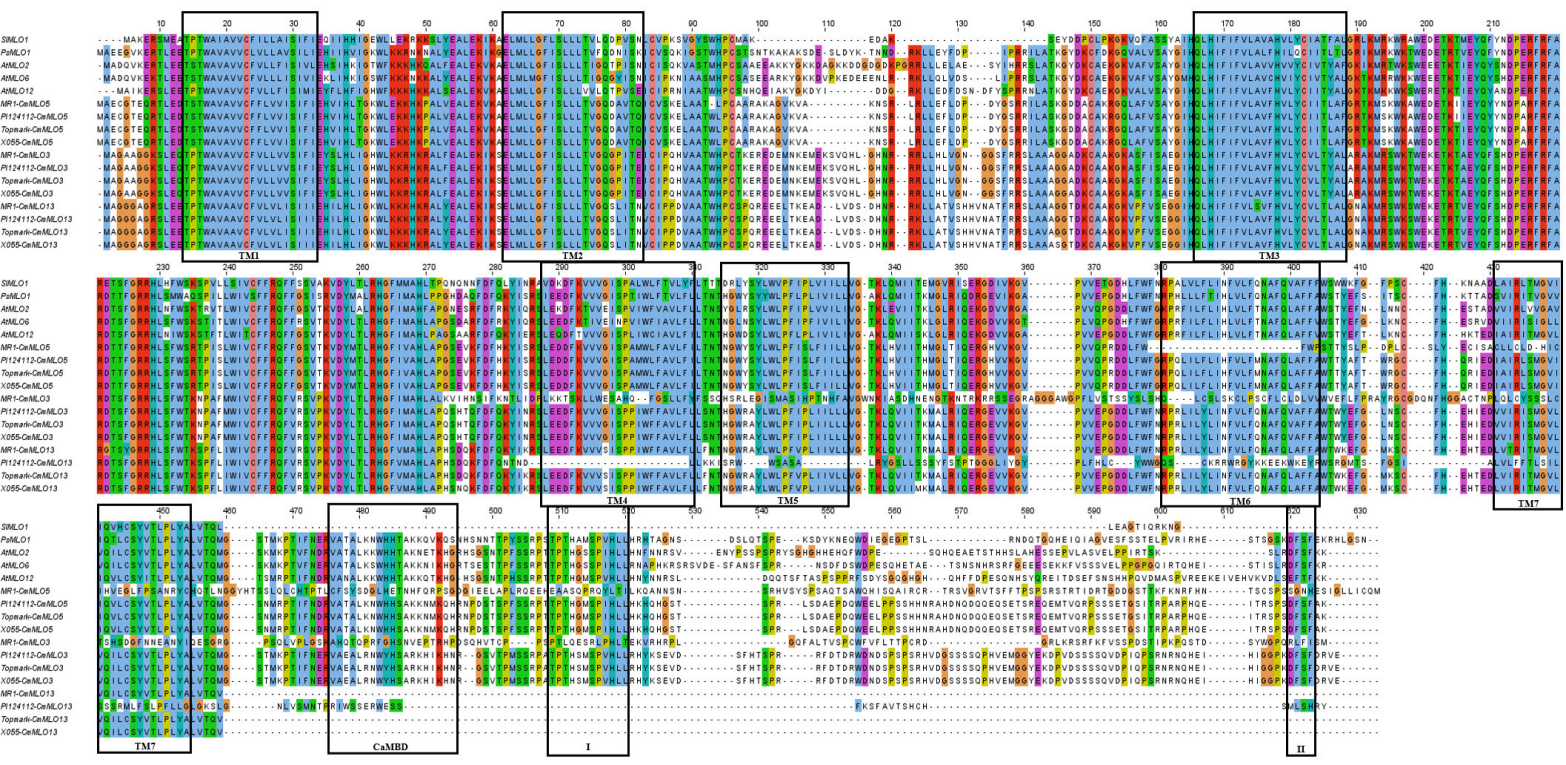
Multi-sequence comparison between the CmMLO protein in Clade III and powdery mildew susceptible MLO proteins in PsMLO1, SIMLO1, along with AtMLO2, 6, and 12. TM1–TM7 is representing seven MLO protein transmembrane domains, and I and II representing two conserved domains in the c-terminal of high polymorphism.

The multiple sequence alignment of the MLO proteins in Clade III (**Figure 7**) revealed that the CmMLO5 and CmMLO3 proteins in the PM-resistant line (MR-1), the CmMLO13 protein in the PM-resistant line (PI124112) had incomplete and altered transmembrane domain position due to base mutations, while all of the remaining MLO proteins contained 7 TM structures. The analysis of CaMDB and the conserved structural domain revealed that CaMDB had approximately 15-20 amino acids away from the 7th transmembrane domain, conserved structural domains I and II are located at the C-terminus of the MLO protein, and structural domain II has the consistent sequence D/E-F-S/T-F. The CmMLO5 and CmMLO3 proteins in the PM-resistant line MR-1, the CmMLO13 protein in the four melon lines, and the SIMLO1 protein do not have a CaMDB with two conserved domains (I and II), but the remaining MLO proteins all contain a CaMDB and two conserved structural domains (I and II).

### Expression characteristics of the *MLO* gene family in melon induced by *P. xanthii race* 1

**Figure 8:**
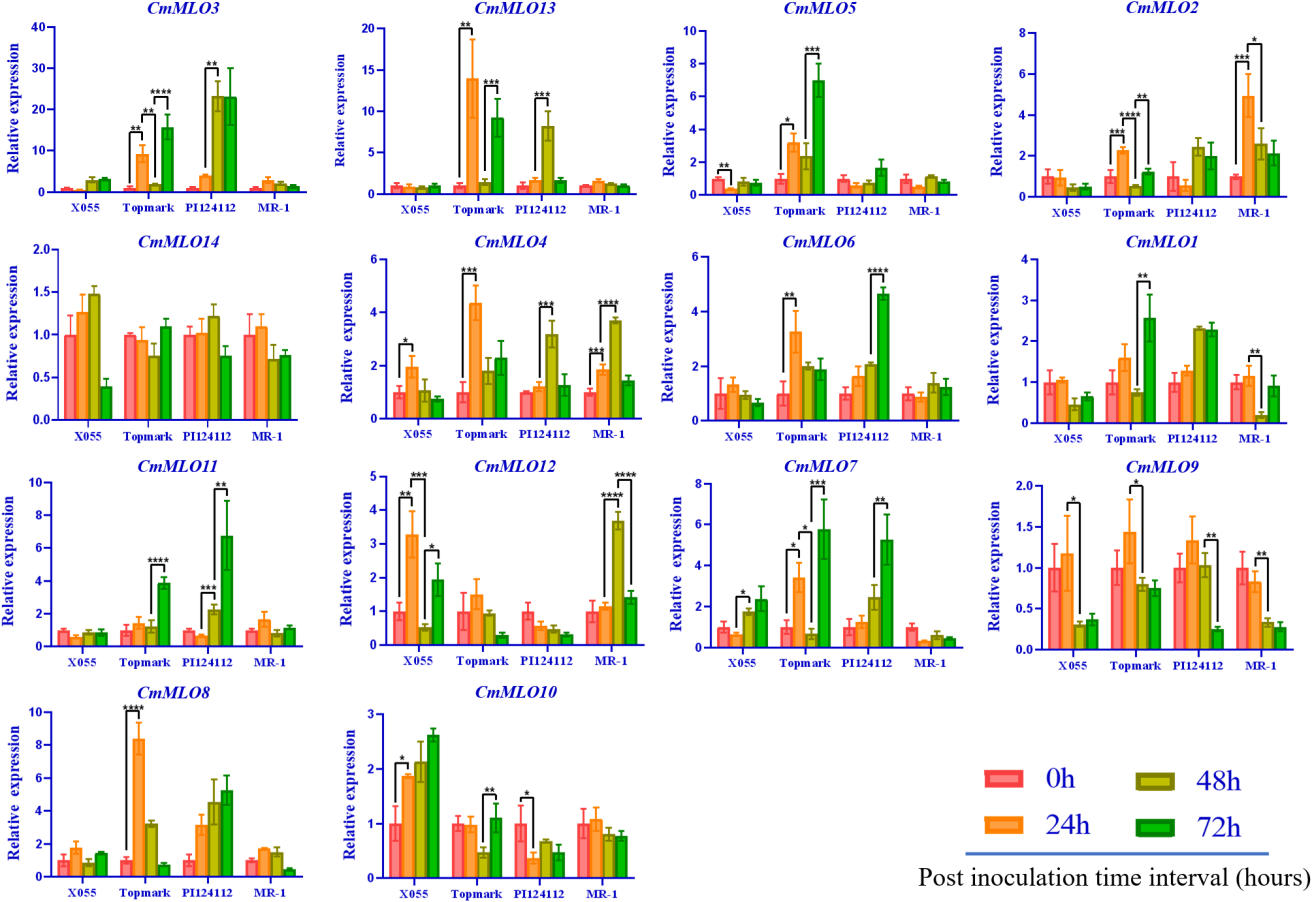
The *MLO* genes expression analysis in different melon materials after infection at different time points. *CmActin* was used as an internal control, samples at 0 h after inoculation were used as controls, and the error line is the standard deviation of three biological replicates. Asterick symbols (*,**,***,****) are representing the significant results at **p < 0.01, ***p < 0.001, ****p < 0.001, and *p < 0.05 level, respectively.

The changes in *CmMLO* gene expression in four different melon lines infected with *P. xanthii* race 1 were analyzed by real-time quantitative PCR (**Figure 8**). We found that the expression changes of 14 *MLO* genes in different resistant and susceptible varieties of melon had great differences after infection with *P. xanthii* race 1. The expression levels of some genes were significantly higher than those of other *MLO* genes in some lines. In addition, significant differences were observed in the expression patterns of different members of the gene family. We found that *CmMLO4* was induced in both PM-susceptible lines at 24 h, while the transcription level was still low at 24 h in resistant materials, and the expression level began to be upregulated at 48 h.

The expression levels of *CmMLO10* and *CmMLO5* were up-regulated at 24 h in a PM-susceptible line, respectively, and no significant change was observed in the two resistant varieties. After infection, nine *CmMLO* genes (*CmMLO7*, *CmMLO8*, *CmMLO12*, *CmMLO6*, *CmMLO13*, *CmMLO3*, and *CmMLO2*) were up-regulated at 24 h in one or two PM-susceptible lines. The expression levels of the other six genes were upregulated at 48 h or 72 h in one of the PM-resistant lines, except for *CmMLO2*; however, the expression trends of these eight genes in the two PM-resistant lines were not completely consistent. In addition, transcription levels of *CmMLO8*, *CmMLO13*, and *CmMLO3* were significantly induced at 24 h in Topmark and 48 h in PI124112. The expression levels of the two lines increased significantly, indicating that the three genes were strongly induced by *P. xanthii* race 1.

After being induced by *P. xanthii* race 1, the expression of *CmMLO3* in the leaves of Topmark and PI124112 was higher than that of the other 13 *CmMLO* genes. At the same time, we also found that the expression level of *CmMLO14* did not change significantly in the four melon lines, and the expression level of *CmMLO9* was down-regulated in the four melon lines after inoculation, suggesting that these two genes may not be induced in the stress response to *P. xanthii* race 1. These results suggest that *CmMLO10* and *CmMLO5* may be genes involved in susceptibility to PM in melon, while *CmMLO8*, *CmMLO4*, *CmMLO13*, and *CmMLO3* may also play a role in PM resistance.

### Multiple sequence alignment analysis

We previously hypothesized that three *MLO* genes (*CmMLO13*, *CmMLO3*, and *CmMLO5*) from Clade III might be involved in the regulation of melon PM. We found that the expression of the *CmMLO5* gene was significantly upregulated in the PM-line Topmark after *P. xanthii* race 1 infection. Therefore, the sequences of the *CmMLO5* gene obtained from the sequencing of the four melon lines (MR-1, PI124112, X055, and Topmark) were compared with the reference genome sequence, and 98.4% homology was found, indicating that *CmMLO5* had been successfully cloned.

**Figure 9:**
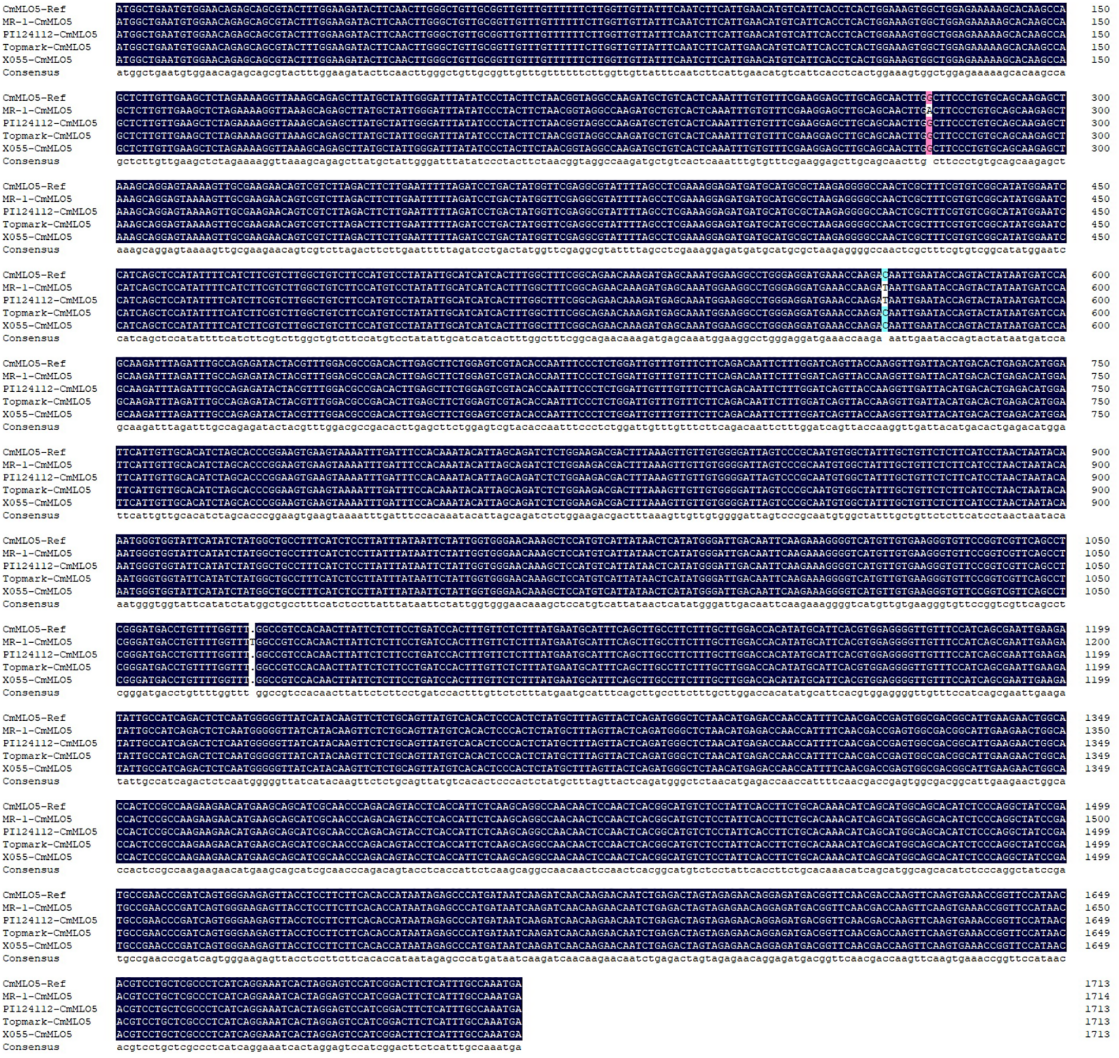
Multiple sequence alignment of the *CmMLO5* gene in four melon lines (MR-1, PI124112, X055, and Topmark), respectively.

According to the multiple sequence alignment (**Figure 9**), there were three base inconsistencies in the nucleotide sequence of the PM-resistant line (MR-1) as compared to the two PM-susceptible lines (X055 and Topmark): a base mutation (G to A) at 279 bp resulting in an amino acid deletion; a base mutation (C to T) at 572 bp resulting in a change of amino acid from T to I; and a base insertion at 1072 bp resulting in a sequence change after amino acid 357^th^, and sequences of conserved motifs of the *CmMLO* gene family was also altered due to base mutations and insertions. The PM-resistant line PI124112 had one base mutation in the nucleotide sequence as compared to the sequences of the PM-susceptible lines (X055 and Topmark), in common with the PM-resistant line (MR-1), a base mutation (C to T) at 572 bp resulted in a change in amino acid from T to I.

## Discussion

In recent years, many studies have shown that the *MLO* gene is significantly associated with PM-resistance in plants, but the in-depth analysis of the *MLO* gene family in melon still remains unclear. In this study, *CmMLO* genes were cloned in four melon lines with different PM-resistances, and the exon-intron composition, lineal homology, and paraphyletic homology of the *MLO* gene family, as well as the changes in expression patterns at different time intervals post-inoculation, were analyzed. A total of 13 *CmMLO* genes, all of which belong to the *MLO* gene family, were cloned from two disease-resistant and two disease-susceptible melon materials. The presence of 14 CmMLO proteins was identified on the basis of the genome-wide gene family analysis, and the absence of the CmMLO14 protein in this study is due to the differences in genetic divergences between the four melon materials and the melon reference genome. The present study results provide a way to sort out how the MLO gene family in melons is related to PM-resistance, and they also give us a theoretical basis for studying its function in more depth.

Further, a total of 14 members of the *CmMLO* gene family were identified by bioinformatic analysis based on melon genome sequencing data. The analysis showed that the conserved motifs and gene structure were different among different members of the *CmMLO* gene family, which further indicated that each member might play different functions. An analysis of the amino acid sites of motifs 1 and 4, two important conserved motifs, showed that leu is the common amino acid site of these two conserved motifs. Leucine may act as a growth regulator or signal molecule used to regulate the biological phenomena and related mechanisms of root growth and morphogenesis (Cheng, 2017). The 3-Methylbdopsyl CoA carboxylase gene is knocked out during Leu degradation, which blocks Leu degradation in mitochondria and affects reproduction in plants (Ding et al., 2012). It has been speculated that *CmMLO* family members containing these conserved motifs may play an important role in promoting growth and development. The chromosomal location analysis showed that the 14 members of the *MLO* gene family were only distributed on eight chromosomes of melon, and the distribution was uneven. There were no *CmMLO* members on four chromosomes: Chr1, Chr2, Chr4, and Chr5. This further indicates the functional diversity of the *CmMLO* gene family.

A phylogenetic tree containing the *MLO* genes from five species was constructed to elucidate the phylogenetic relationships *MLO* family members between *C. melo* and other plants. In total, six different clades (I to VI) were identified (**Figure 6**), and these clades were named on the basis of classification as earlier reported (Devoto et al., 2003). The phylogenetic analysis divided the *CmMLO* genes into five different clades (**Figure 6**), which was consistent with the structure, evolution and functional inference on the *MLO* gene family in three cultivated Cucurbitaceae spp as previously reported (Iovieno et al., 2015). The phylogeny analysis showed that the *CmMLO* genes were distributed in five of the six clades. Among them, Clade III includes some *MLO* genes from dicotyledonous plants; these genes were previously determined to be related to PM-susceptibility (Consonni et al., 2006; Bai et al., 2008; Pavan et al., 2011). In Clade III, three *CmMLO* genes (*CmMLO13*, *CmMLO3*, and *CmMLO5*) were clustered with *AtMLO2*, *AtMLO6* and *AtMLO12*, *SlMLO1*, and *PsMLO1*. In addition, the amino acid sequence of the identified *MLO* gene was compared and the conserved sequence of different genes in *Arabidopsis*, tomato, pea, and melon in Clade III were studied. It was found that the three *CmMLO* genes were highly conserved in the predicted TM domain location (Devoto et al., 2003). In addition, CaMBD at the C-terminal of the MLO protein was found to be highly conserved in the entire *Arabidopsis MLO* family (Kim et al., 2002) among the eight different *MLO* gene members analyzed in the above plants. Panstruga and co-researchers also found two other conserved regions (I and II) at the C-terminal of the MLO protein that play an important role in regulating PM infection (Panstruga, 2005b). The peptide domain I is located about 15–20 residues downstream of CaMBD and is characterized by conserved serine and threonine residues. The peptide domain II is located distal to the C-terminal and contains the consistent sequence D/E-F-S/T-F. In this study, the C-terminus of six MLO proteins has these two conserved domains (**Figure 7**). All of these findings suggest that MLO proteins containing CaMBD and peptide domains I and II are potentially functionally conserved.

According to earlier research findings, *MLO* mutant plants could inhibit the formation of haustrium and epidermal cells infected by the pathogen that did not die immediately (Koga et al., 1990; Nelson et al., 1990; Aist and Bushnell, 1991). In addition, in the broad-spectrum resistance response mediated by the *MLO* gene, the cell wall is thickened by regulating the occurrence of a mastoid reaction to resist the primary infection of PM (Peterhansel et al., 1997). Some researchers have proven through validated experiments that mastoid resistance is controlled by the *MLO* gene. Therefore, the mastoid reaction provides plants with the main defense against pathogen infection of host cells in the early stage of *MLO*-mediated resistance (Aist, 2003). Given that the putative functions of these *MLO* genes in Clade III are derived from *S. lycopersicum*, *P. sativum*, and *A. thaliana*, we infer that this clade is important for *C. melo* because these genes are necessary for PM susceptibility (Consonni et al., 2006; Bai et al., 2008; Pavan et al., 2011). Thus, the clade may be unique to dicotyledons. The *MLO* gene was previously confirmed to be a gene related to PM sensitivity and the expression level of *this* gene in PM-susceptible plants was rapidly up-regulated after PM infection (Cheng et al., 2013). Herein, we concluded that *CmMLO* genes are associated with melon PM susceptibility and may be significantly up-regulated in susceptible lines following PM infection. The changes in *CmMLO* gene expression in four different melon lines infected with PM were analyzed by real-time quantitative PCR, and we found that the *CmMLO5* gene of Clade III was significantly up-regulated in the susceptible line (Topmark) at 24h and 72h after infection by PM. In combination with the microscopic observation of four periods (0h, 24h, 48h and 72h) of Trypan Blue staining of Topmark leaves of susceptible lines after *P. xanthii* race 1 infection, we hypothesize that the *CmMLO5* gene plays an important role in both periods when the PM pathogen haustorium invades the leaf cells and during hyphal differentiation to produce conidiophores.

Multiple sequence alignment analysis of the *CmMLO5* gene in four melon lines showed that both PM resistant lines (MR-1 and PI124112) showed a single base mutation (C to T) at 572 bp compared to two PM susceptible lines (X055 and Topmark), which resulted in a change in amino acid from T to I. So we speculate that this single base mutation (C to T) at 572 bp, which further results in the loss of protein function and thus confers resistance to PM in melon. However, the expression level of the *CmMLO5* gene in the PM-susceptible line (X055) did not change significantly. This may be caused by the different disease-susceptible mechanisms of different melon lines. In a recent molecular study, a genetic mapping population was developed, and mapping of the recessive gene *CmPMrs* (*MELO3C012438*) was done using a forward genetics mapping strategy, which revealed that stem resistance on melon is mainly controlled by a candidate genetic region positioned on chromosome 10 (Cui et al., 2022). This evidence could also suggest that the *CmMLO5* gene is associated with PM susceptibility.

The specific reasons need to be further tested for verification. The expression level of *CmMLO10* in Clade VI showed up-regulation in susceptible lines after the inoculation, which was consistent with the results of earlier reported study (Dong et al., 2019), but no significant change was observed in the expression level in resistant lines. This may occur because the *MLO* gene is a gene that negatively regulates plant disease resistance (Piffanelli et al., 2002; Kumar et al., 2016), so its expression level in susceptible lines is significantly up-regulated after being infected by *P. xanthii* race 1. However, a great difference was observed in the variation of different PM lines, which inferred that different genes in different varieties had different sensitivities to PM invasion. Mutants of *AtMLO4* and *AtMLO11* in the same clade as *CmMLO10* can regulate asymmetrical root growth (Chen et al., 2006). So, it is thought that *CmMLO10* may also control root growth in addition to making plants resistant to PM.

Further, the expression levels of the *CmMLO8*, *CmMLO13*, and *CmMLO3* genes in resistant and susceptible lines changed significantly, after the induction of *P. xanthii* race 1 infection. In addition, the range of variation in gene expression of *CmMLO3* in the two varieties was larger than that of other genes. After *P. xanthii* race 1 infection, the expression levels of these three genes increased in both PM-resistant and PM-susceptible lines, but the response time was different. However, the up-regulation of *MLO* gene expression in PM-resistant lines may be caused by biological or abiotic stress (Howlader et al., 2017), and the specific reasons need to be further verified by future experiments. After infection with *P. xanthii* race 1, the expression levels of *the CmMLO14* gene at the other three time points in the four lines showed no significant change compared with that at 0 h, indicating that the *CmMLO14* gene was not induced by *P. xanthii* race 1. The expression of the *CmMLO9* gene was down-regulated in the four melon lines at 48 h and 72 h. This may occur because of the inhibition of *CmMLO9* gene expression during the interaction between melon PM and melon, and this type of inhibition does not change due to the natural resistance and susceptibility of melon varieties. So, it is speculated that the *CmMLO9* gene is not involved in the regulation of PM in melon. However, the minimization of the expression level may be caused by other factors or pathogenic bacteria infecting melon indirectly, so it has no direct relationship with pathogenic bacteria. In addition, although two pairs of fragmented genes (*CmMLO6* and *CmMLO4*, and *CmMLO13* and *CmMLO2*) have similarities in their sequences, their expression patterns seem to be different, which indicates that the repeated genes of melon have achieved functional differentiation during the period of evolution.

## Conclusion

The current study demonstrated a genome-wide bioinformatics analysis for the identification of the *CmMLO* gene family and found that the amino acid sequence of the *CmMLO* gene shows significant conserved protein characteristics. By cloning the *CmMLO* gene sequence in different melon lines, analyzing the *CmMLO* gene expression pattern after infection, and microscopic observations of the infestation pattern of PM, we concluded that the *CmMLO5* (*MELO3C012438*) gene plays a negative role in regulating PM-resistance in the susceptible line (Topmark). We also assumed that this single base mutation (C to T) at 572 bp further results in the loss of protein function and thus confers resistance to PM in melon. In short, this study is the first step toward figuring out how the *CmMLO* gene family works, which could provide a certain basis for molecular and functional research in an important direction in the future.

## Data availability statement

The original contributions presented in the study are included in the article/**Supplementary Material**. Further inquiries can be directed to the corresponding authors.

## Author contributions

TZ and NX performed the bioinformatic analysis and molecular experiment. SA assisted in the data curation, formal analysis, and scientific writing of the manuscript. SA and PG are the corresponding authors who performed conceptualization, review, and editing of the manuscript. All authors contributed to the article and approved the submitted version.
