# Supplementary material for: Genome-wide identification, evolution, and expression analysis of MLO gene family in melon (Cucumis melo L.)
Source: Front Plant Sci. 2023 Feb 24;14:1144317. doi: 10.3389/fpls.2023.1144317 (PMC9998560; doi:10.3389/fpls.2023.1144317)
Supplement: Supplementary file 1 [file DataSheet_1.zip › Supplementary Material/Supplementary Figures-alter.docx]

**Supplementary Figures**

**Supplementary Figure S1 |** Multiple sequence alignment of total 13 *CmMLO* genes in four melon lines (MR-1, PI124112, X055, and Topmark).

*CmMLO1* Gene

*CmMLO2* Gene

*CmMLO3* Gene

*CmMLO4* Gene

*CmML05* Gene

*CmMLO6* Gene

*CmMLO7* Gene

*CmMLO8* Gene

*CmMLO9* Gene

*CmMLO10* Gene

*CmMLO11* Gene

*CmMLO12* Gene

*CmMLO13* Gene
