# Supplementary material for: Genome-wide identification, evolution, and expression analysis of MLO gene family in melon (Cucumis melo L.)
Source: Front Plant Sci. 2023 Feb 24;14:1144317. doi: 10.3389/fpls.2023.1144317 (PMC9998560; doi:10.3389/fpls.2023.1144317)
Supplement: Supplementary file 1 [file DataSheet_1.zip › Supplementary Material/Supplementary Tables.docx]

**Supplementary Table**

**Supplementary Table S1 |** The information of *CmMLO* genes cloning primers used in this study.

| **Gene** | **Primer sequence (5’-3’)** |
| --- | --- |
| *CmMLO1* | F: ATGGGCGGCGGAGGTGAA  R: CTTTTTGGCACAATAACCAGCCTGT |
| *CmMLO2* | F: ATGGCCGCCGCAGCCTTAG  R: TTACTTTTCTTTGAACTTGGCTCTT |
| *CmMLO3* | F: ATGGCGGGGGCAGCCG  R: TCATTCAACTCTATCAAATGAAA |
| *CmMLO4* | F: ATGTTTCTGGTTGTTTATTATTTG  R: TCATTTTGTTGGTCCAGGTT |
| *CmMLO5* | F: ATGGCTGAATGTGGAACAGAGCA  R: TCATTTGGCAAATGAGAAGTCCGA |
| *CmMLO6* | F: ATGTCTGTTTTTTGTCTTTGCTTCT  R: TTATCCTAGTGGTGCAGGCTTTATA |
| *CmMLO7* | F: ATGCTCTTTGGACTTCTTTCGTTGT  R: TCAATCATCCTCATTGCCTTCAC |
| *CmMLO8* | F: ATGGCTGAAAATGCCTCCC  R: TTATCTCGGCAAAGATGAGCTTC |
| *CmMLO9* | F: ATGGAACTTCAAGGAGGAAGG  R: TCATGTCCTTTTGAACAAGTCG |
| *CmMLO10* | F: ATGGCTGAAAATGAACAGGAG  R: CTAGGTGTTGTTCTGGCTCGA |
| *CmMLO11* | F: ATGCTTTTAGGGAATAAGGTGC  R: TTAATGAGTTGATTTGGAGTC |
| *CmMLO12* | F: ATGGATGGAAGAGGGAAT  R: TTATTTAGTTGAGGCATT |
| *CmMLO13* | F: ATGGCCGGAGGTGGCG  R: CTATACCTGTGTGACTAGAGCAT |
| *CmMLO14* | F: ATGGGTGGCGGTGGTGGT  R: TCAACTCAACAAGTCACGATTAGCA |

**Supplementary Table S2 |** The information of *CmMLO* genes primers used for real-time PCR primer sequences.

| **Gene** | **Primer sequence( 5’-3’)** |
| --- | --- |
| *CmMLO1* | F:TCGTCAACGACGAAGCATTT  R:TGAAGCAGATCCCTCAGCAA |
| *CmMLO2* | F:GCATTCCCATCACCATGACC  R:GTTCGGCTACTACCCTCGAA |
| *CmMLO3* | F:TGTCACCTGTCCACCTTCTC  R:GGTGAGGGCGAATCATTGTC |
| *CmMLO4* | F:AGATTCGAGGTTGGAAGGCA  R:TCAGCCTTACCAACGGACTT |
| *CmMLO5* | F:TTGGCTTTCGGCAGAACAAA  R:CGGCGTCCAAACGTAGTATC |
| *CmMLO6* | F:CCGTCCATTGCAAACTCCAT  R:TGTGACCTCCAGCCAATGAT |
| *CmMLO7* | F:CTCGTTTGGCTGCTCTGTTT  R:AAAGCATTCTTGTGGCAGCA |
| *CmMLO8* | F:ATGATGCCACAATCCACACG  R:TAACAGAGGCGGACCCATTT |
| *CmMLO9* | F:CCATGGAGCAGGAGTCGTAT  R:TCGAACCACGTACTTGTGGA |
| *CmMLO10* | F:TTGGTCTGTTGCTTCTGTGC  R:CTGCCTCAAACAGTGGCTTT |
| *CmMLO11* | F:GCTCTACGCCATTGTCACTC  R:GACTCCTTGACTGGAGCCAT |
| *CmMLO12* | F:TGGAGAAACCTGCCTACATCA  R:CTTCTGCATCAGGTGGCTTC |
| *CmMLO13* | F:GTTCGACGATGAAGCCTACG  R:ATGTGTTTACGAGCCGTGTG |
| *CmMLO14* | F:CTCCTCTGCTATGGGCATCA  R:GGAATCCCTTGAACCACTGC |
